# Effect of position management combined with active cycle of breathing techniques on reducing postoperative pulmonary complications in lung cancer patients

**DOI:** 10.1186/s12885-025-15029-4

**Published:** 2025-11-18

**Authors:** Guo Ning, Chen Sihan, Ji Daihong, Zhao Zhilong, Zhao Aihua, Li Liting, Gao Heling

**Affiliations:** 1 Affiliated Zhongshan Hospital of Dalian University, Dalian, 116001 China; 2 Chifeng Municipal Hospital, Chifeng, 024099 China

**Keywords:** Position management, Active cycle of breathing techniques, Lung cancer, Pulmonary complication

## Abstract

**Objective:**

To explore the effect of position management combined with active cycle of breathing techniques (ACBT) in reducing postoperative complications in patients with lung cancer.

**Methods:**

Between March 2022 and March 2023, 213 patients undergoing thoracoscopic radical lung cancer surgery at a tertiary hospital in Dalian were screened and randomized into three groups: control (routine nursing, n=71), experimental group 1 (routine nursing + ACBT, n=73), and experimental group 2 (routine nursing + ACBT + position management, n=69). After excluding 6 patients, 207 were analyzed (69 per group). Outcomes compared included incidence of postoperative pulmonary complications, duration of chest tube placement, hospital stay, postoperative oxygen saturation (SpO₂), and nursing satisfaction.

**Results:**

Postoperative pulmonary complications occurred in 23.2% (17/69) of the control group, 13.0% (9/69) of experimental group 1, and 4.3% (4/69) of experimental group 2 （*X2, p=0.007*）. Repeated measures ANOVA showed significant differences in SpO₂ across groups (*p* < 0.001), time points (*p*< 0.001), and their interaction (*p* < 0.001) on postoperative days 1–5. The average drainage tube duration was 3.74 ± 2.83 days (control),3.90 ± 3.25 days (group 1), and 2.74 ± 1.11 days (group 2) (*H*=10.638, *p*=0.005). The postoperative hospital stays were 6.42 ± 3.76 days (control), 6.39 ± 4.33 days (group 1), and 4.99 ± 1.24 days (group 2) (*H*=7.868, *p*=0.020). Satisfaction scores were 4.46 ± 0.50 (control), 4.66 ± 0.48 (group 1), and 4.87 ± 0.34 (group2) (*H*=26.121, *p*=0.000).

**Conclusions:**

Position management combined with active cycle of breathing techniques (ACBT) may promote respiratory secretion clearance, potentially reduce postoperative pulmonary complications, shorten chest tube duration and hospitalization, and improve oxygen saturation and patient satisfaction in lung cancer patients. These findings suggest potential clinical benefits of the combined approach in perioperative nursing for lung cancer patients; however, larger multicenter trials with longer follow-up are required to validate these results.

## Background

According to statistics, lung cancer has the highest incidence and mortality rates in most countries, severely threatening human health [1]. With advances in medical technology, thoracoscopic surgery has become the primary treatment for lung cancer [2, 3]. However, after surgery, patients often experience reduced Lung capacity and limited diaphragm movement. They may also develop structural changes in the Lungs and capillaries. Surgical site pain restricts effective coughing, sputum clearance, and deep breathing. These factors contribute to the accumulation of respiratory secretions, significantly increasing the risk of postoperative pulmonary complications. The incidence of such complications can be as high as 40%, which prolongs hospitalization, increases patient discomfort and financial burden, and leads to inefficient use of healthcare resources [4, 5]. Therefore, Promoting the effective expulsion of respiratory secretions after surgery is essential to prevent pulmonary complications [6]. Currently, common methods include coughing postural drainage, and mechanical suction. However, these methods have limitations. Some patients cannot cough effectively, percussion techniques may be inconsistent or difficult to standardize, and repeated suctioning can cause discomfort or even injury [7–9]. The Active Cycle of Breathing Technique (ACBT) is a short-term, patient-controlled rehabilitation method that has been shown to improve airway clearance, enhance sputum expectoration, and support lung function [10]. Position management is another simple, low-cost, and effective nursing strategy that can help remove secretions without causing patient distress. It also plays a preventive role against pulmonary infections [11–13]. Combining ACBT with position management enables position adjustments based on airway secretions. This approach is time-efficient, allows independent or assisted training, and effectively promotes secretion clearance [14]. This approach promotes the rapid recovery of early postoperative lung function, reduces postoperative respiratory complications such as pulmonary infections, atelectasis, and hypoxemia, and provides a theoretical basis for early removal of the chest closed drainage tube. This can also reduce the average length of hospital stay for lung cancer surgery patients and improve patient satisfaction. Therefore, this study reports the nursing intervention of position management combined with ACBT, compared with ACBT and routine nursing care, for patients undergoing thoracoscopic radical lung cancer.

## Objects and methods

### Study subjects

**Fig. 1:**
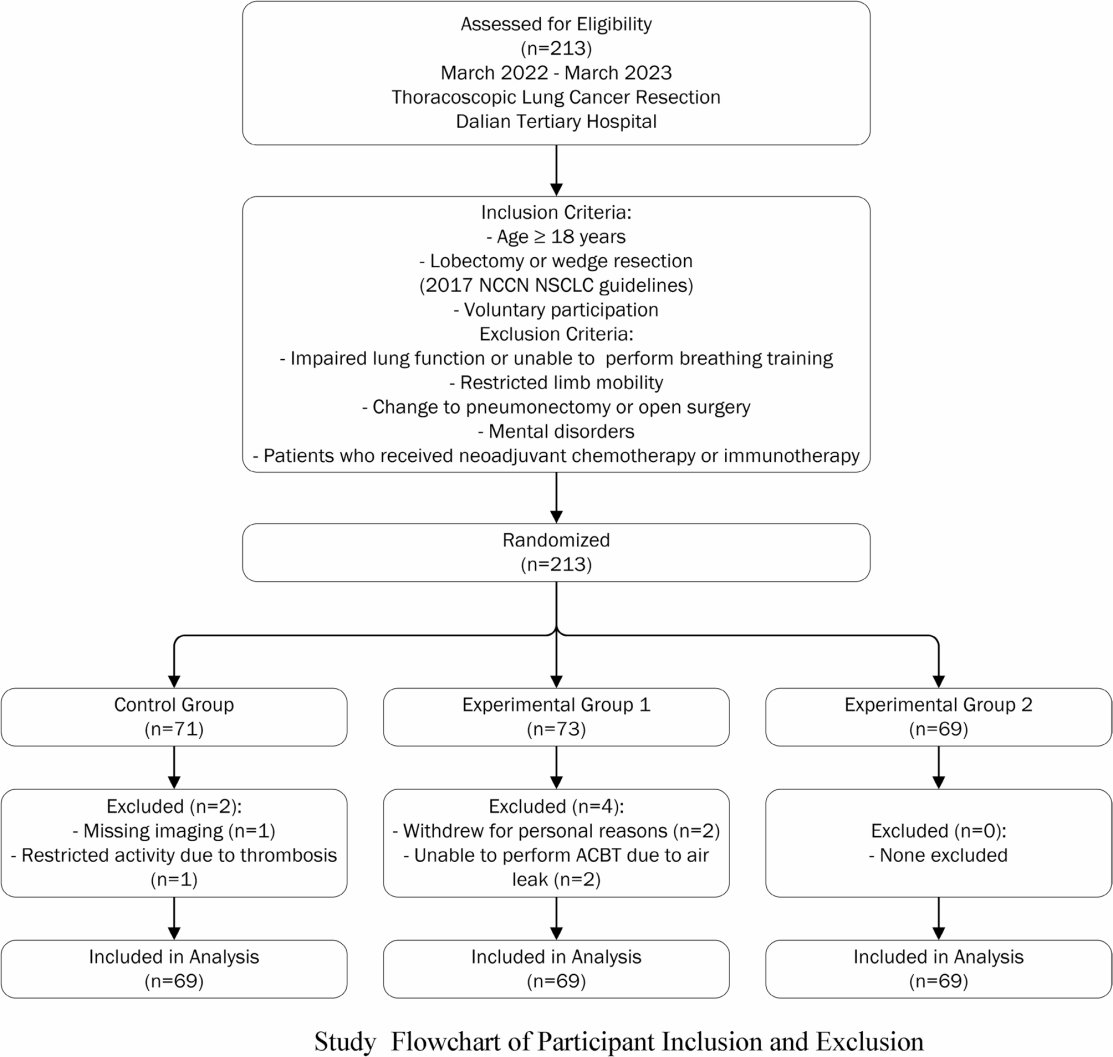
Study flowchart of participant inclusion and exclusion

Between March 2022 and March 2023, 213 patients undergoing thoracoscopic radical lung cancer surgery at the Department of Thoracic Surgery in a tertiary hospital in Dalian were recruited via convenience sampling. Participants were randomized into three groups—control (*n* = 71), experimental group 1 (*n* = 73), and experimental group 2 (*n* = 69)—using a random number table. Following the application of exclusion criteria, 6 patients were excluded (2 from the control group, 4 from experimental group 1, and none from experimental group 2), resulting in 207 patients (69 per group) included in the final analysis. Inclusion criteria were: (1) age ≥ 18 years; (2) diagnosis of non-small cell lung cancer (NSCLC) with planned lobectomy or wedge resection, per the 2017 National Comprehensive Cancer Network (NCCN) guidelines; and (3) voluntary participation. Exclusion criteria included: (1) lung conditions precluding respiratory training; (2) limited limb mobility preventing position management; (3) intraoperative conversion to total pneumonectomy or open surgery; (4) mental disorders hindering cooperation; and (5) receipt of neoadjuvant chemotherapy or immunotherapy. The participant flow is detailed in Fig. 1. Study Flowchart of Participant Inclusion and Exclusion. This study was approved by the hospital’s ethics committee (Ethical Review Opinion No. 2022001), and all participants provided written informed consent.

### Methods

#### Establishment of the research team

The research team consisted of 9 members: 1 chief thoracic surgeon, 1 attending thoracic surgeon, 1 head thoracic nurse (with over 20 years of experience), 1 rehabilitation therapist, 4 thoracic nurses, and 1 master’s student. All members held at least a bachelor’s degree. Except for the master’s student, all had more than 5 years of work experience. Both the attending physician and head nurse held valid National GCP certificates. Task Distribution: The chief thoracic surgeon and the head nurse were responsible for overall coordination, quality control, and guidance. Their responsibilities included establishing the research team, conducting training, and supervising the development and implementation of the intervention combining position management with Active Cycle of Breathing Techniques (ACBT). The attending physician, rehabilitation therapist, and thoracic nurses carried out the intervention plan. The attending physician, rehabilitation therapist, and thoracic nurses carried out the intervention plan. The master’s student assisted in literature review, intervention plan development, and was responsible for data collection, organization, and analysis.

#### Intervention methods

##### Control group

Patients received routine treatment and nursing care. They were guided to perform deep breathing exercises three times per day, for 15–30 min each session, continuing for 5 days after surgery.

##### Experimental group 1

In addition to routine care, patients practiced ACBT. The ACBT protocol included three components:

- Breathing Control (BC): Starting 6 h after surgery, patients chose a comfortable position (semi-recumbent, sitting, or standing). They inhaled through the nose and exhaled through the mouth (with or without pursed lips). Hands were placed on the abdomen. The abdomen rose during inhalation. This was performed slowly for 3–4 breaths.
- Thoracic Expansion Exercises (TEE): Patient placed their hands on their chest. Patients inhaled nasally, held their breath for 3 s, and exhaled orally. The chest expanded during inhalation and contracted during exhalation. This was repeated 3–4 times, followed by FET or return to BC.
- Forced Expiration Technique (FET): Patients placed their hands in front, mimicking holding a mirror. They inhaled through the nose, held their breath for 3 s, and exhaled forcefully with a “ha” sound to “fog” the imaginary mirror. This was repeated 2–3 times.

The full ACBT cycle was performed three times Daily for 15–30 min each session, continuing for 5 days.

##### Experimental group 2

Patients in this group received position management in combination with ACBT, based on the control group protocol. Position management included:

- Initial postoperative positioning (0–6 h): Patients were placed in a head-down, flat-lying position with the head turned to one side and the bed head raised to 30–45°.
- After 6 hours: During waking hours, patients rested in a semi-sitting position with the head of the bed raised to 60°. During sleep, the bed head was raised to 15–30°, adjusted for comfort.
- Other positions:
  1. Sitting or standing with a forward-leaning posture to assist diaphragm movement and improve lung volume.
  2. Postural drainage for retained sputum based on the surgical site.
  3. Prone positioning (if tolerated) to improve oxygenation and ventilation-perfusion (V/Q) ratio.

ACBT procedures for this group were the same as those in Experimental Group 1

### Specific protocol for combined position management and ACBT

Postural adjustments and ACBT were tailored based on each patient’s surgical procedure and airway secretion status.

- Within the first 6 h after surgery: patients received only position management. Before full anesthesia recovery, they were assisted into a head-down, flat-lying position with the bed head elevated to 30–45°. to 30–45°.

- After anesthesia recovery (same day): patients were guided into a semi-recumbent position. ACBT began 6 h after surgery. For patients undergoing wedge resection or lobectomy, during waking hours, the preferred positions were on the healthy side with the bed raised to 60° or in a seated position.

- During mobilization: patients stood with the trunk slightly bent forward. For those with excessive sputum, poor coughing ability, or ineffective expectoration, prone positioning (with the head turned to one side) was used, and ACBT was adjusted to their respiratory tolerance.

### Training frequency and duration

**Fig. 2:**
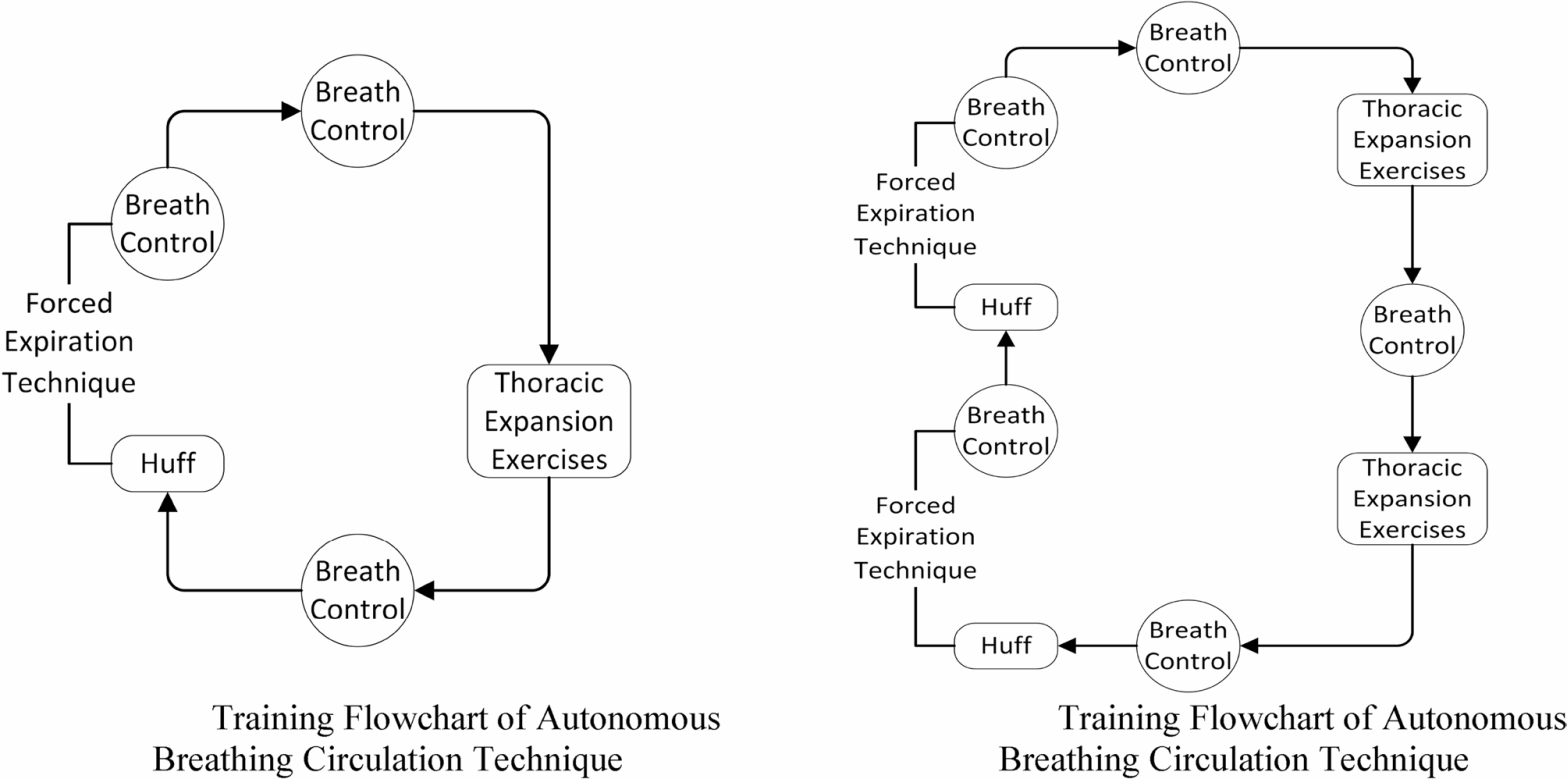
Training flowchart of autonomous breathing circulation technique

All patients received ACBT three times a day—for 15–30 min each session—for 5 consecutive days. During training, patients either sat or stood and performed BC, TEE, and FET according to tolerance. The inhalation volume was calculated based on a tidal volume of 10 mL/kg plus compensatory volume. Patients were instructed to hold their breath for 3–5 s after inhalation and to observe for bubbling in the chest drainage bottle. If no abnormalities were observed, the training proceeded as planned Fig. 2.

#### Observation indicators

##### (1) Incidence of pulmonary complications

This includes cases of pulmonary infection and atelectasis.

- Pulmonary infection is diagnosed by two team physicians using the following criteria:
  - Imaging shows signs of lung infection.
  - Clinical symptoms such as fever > 38 °C.
  - White blood cell count > 10 × 10⁹/L and neutrophils > 10%.
  - Presence of purulent secretions.
  - Antibiotics are initiated or continued based on clinical judgment.

A diagnosis is confirmed when at least two of the five criteria are met.

- Atelectasis is evaluated based on chest X-ray findings, patient symptoms, lab results, and observations of leakage in the chest drainage bottle. The incidence rates of postoperative pulmonary complications will be compared among the three groups.

##### (2) Oxygen saturation (SpO₂)

The average daily SpO₂ levels from postoperative days 1 to 5 will be compared among the three groups.

##### (3) Drainage tube retention and hospital stay duration

Drainage tube retention days refer to the number of days from surgery to chest tube removal. Hospital stay duration refers to the number of days from the first postoperative day to discharge.

##### (4) Patient satisfaction with rehabilitation care

Satisfaction will be assessed using a 5-point Likert scale:

- 1 = Very dissatisfied.
- 2 = Dissatisfied
- 3 = Neutral
- 4 = Satisfied
- 5= Very satisfied

Higher scores indicate greater patient satisfaction.

#### Data collection

General patient information will be collected from the medical records after obtaining informed consent. Data on pulmonary complications, drainage tube retention, and hospital stay duration will be collected from medical records after discharge. SpO₂ levels will be measured daily by trained nurses after breathing exercises. Three measurements will be taken each day, and the daily average will be recorded. Patient satisfaction will be assessed before discharge. Two dedicated researchers will manage data organization, verification, and analysis.

#### Statistical methods

All data were analyzed using SPSS 23.0. A significance level of α = 0.05 was used, with *P* < 0.05 was considered statistically significant. Descriptive statistics were used for general data. Normally distributed continuous variables are expressed as mean ± standard deviation. Categorical variables are presented as frequencies and percentages. Comparisons among the three groups were performed using ANOVA, chi-square tests, or rank-sum tests, depending on the data type.

## Results

### Baseline characteristics of the participants

A total of 213 patients undergoing thoracoscopic lung cancer resection (March 2022 - March 2023) at a tertiary hospital in Dalian were assessed for eligibility and randomized into three groups (control: *n* = 71, experimental group 1: *n* = 73, experimental group 2: *n* = 69) using a random number table. After excluding 6 patients (2 from the control group due to missing imaging [*n* = 1] and restricted activity due to thrombosis [*n* = 1]; 4 from experimental group 1 due to personal withdrawal [*n* = 2] and inability to perform ACBT due to postoperative air leak [*n* = 2]; none from experimental group 2), 207 patients were included in the analysis, with 69 patients in each group. There were no statistically significant differences in baseline characteristics—such as age, gender, ethnicity, marital status, occupational status, lung cancer stage, smoking, body mass index, tumor type, and surgical method—among the three groups (all *P* > 0.05) (Table 1). The participant flow, including screening, randomization, exclusion, and analysis, is illustrated in Fig. 1. Study Flowchart of Participant Inclusion and Exclusion.

**Table 1:** Comparison of general data among the three groups (*N* = 207)

Item	Control Group	Experimental Group 1	Experimental Group 2	Statistic Value	*P*
Number of cases (n)	69	69	69		
Age (years, ±s)	63.10 ± 11.63	61.73 ± 11.91	60.88 ± 11.26	0.642^a^	0.527
Gender [n (%)]				3.042^b^	0.218
Male	22 (31.9)	27 (39.1)	32 (46.4)		
Female	47 (68.1)	42 (60.9)	37 (53.6)		
Ethnicity [n (%)]				1.592^b^	0.451
Han	64 (92.8)	67 (97.1)	64 (92.8)		
Minority	5 (7.2)	2 (2.9)	5 (7.2)		
Marital Status [n (%)]				0.538^b^	0.585
Married	62 (92.8)	61 (88.4)	61 (88.4)		
Divorced	2 (2.9)	3 (4.3)	2 (2.9)		
Widowed	3 (4.3)	5 (7.2)	6 (8.7)		
Occupational Status [n (%)]				0.128^b^	0.880
Unemployed	11 (16.0)	14 (20.3)	13 (18.8)		
Employed	19 (27.5)	18 (26.1)	19 (27.6)		
Retired	39 (56.5)	37 (53.6)	37 (53.6)		
Lung Cancer Stage [n (%)]				1.990^b^	0.139
Stage I	65 (92.8)	56 (81.2)	61 (88.4)		
Stage II	3 (4.3)	7 (10.1)	4 (5.8)		
Stage III	2 (2.9)	6 (8.7)	4 (5.8)		
Smoking [n (%)]				2.223^b^	0.329
Smoker	6 (8.7)	12 (17.1)	10 (14.5)		
Non-smoker	63 (91.3)	57 (82.9)	59 (85.5)		
Body Mass Index (± s)	23.99 ± 2.97	24.82 ± 2.80	24.70 ± 3.31	1.531^a^	0.219
Tumor Type [n (%)]				0.182^b^	0.913
Adenocarcinoma	66 (95.7)	65 (94.2)	66 (95.7)		
Squamous Cell Carcinoma	3 (4.3)	4 (5.8)	3 (4.3)		
Surgical Method [n (%)]^c^				0.149^b^	0.928
Lobectomy	65 (92.8)	63 (91.3)	63 (91.3)		
Wedge Resection	5 (7.2)	6 (8.7)	6 (8.7)		

Note: ^a^represents ANOVA; ^b^represents Chi-square test；^c^represents no patients in any group received preoperative chemotherapy or immunotherapy

### Postoperative pulmonary complications

There was a statistically significant difference in the incidence of postoperative pulmonary complications among the three groups (*χ²* = 10.058, *P* = 0.007). Similarly, the incidence of atelectasis differed significantly (*χ²* = 7.045, *P* = 0.030). However, no significant difference was observed in the incidence of postoperative pulmonary infections (*χ²* = 2.911, *P* = 0.233) (Table 2).

**Table 2:** Comparison of postoperative pulmonary complications among the three groups

Group	Number of Cases (*n*)	Atelectasis [*n* (%)]	Pulmonary Infection [*n* (%)]	Pulmonary Complications [*n* (%)]
Control Group	69	10 (14.49)	7 (10.1)	17 (23.2)
Experimental Group 1	69	4 (5.8)	5 (7.2)	9 (13.0)
Experimental Group 2	69	2 (2.9)	2 (2.9)	4 (4.3)
*χ²*	-	7.045	2.911	10.058
*P* Value	-	0.030*	0.233	0.007*

*p < 0.05

### Postoperative oxygen saturation (SpO₂) groups

SpO₂ values on postoperative days 1 to 5 were analyzed using repeated-measures ANOVA. Mauchly’s test indicated a violation of the sphericity assumption (*P* = 0.000), so multivariate testing was used.

Results showed significant differences in SpO₂ across time points (*P* < 0.05) and between groups (*P* < 0.05), with a significant time–group interaction effect (*P* < 0.05) (Table 3).

**Table 3:** Repeated measures ANOVA of postoperative blood oxygen saturation among the three groups

	Control Group (Mean ± SD)	Experimental Group 1 (Mean ± SD)	Experimental Group 2 (Mean ± SD)	Between Group Comparison F Value	Between Group Comparison *P* Value
Postoperative Day 1	95.13 ± 1.54	96.75 ± 1.25	97.81 ± 0.75	83.569	0.000*
Postoperative Day 2	96.07 ± 1.16	97.25 ± 1.46	98.80 ± 0.66	99.356	0.000*
Postoperative Day 3	96.65 ± 1.10	97.35 ± 1.28	98.46 ± 0.677	52.374	0.000*
Postoperative Day 4	98.1 ± 0.789	98.42 ± 0.63	98.59 ± 0.67	8.814	0.000*
Postoperative Day 5	98.45 ± 0.78	98.59 ± 0.63	98.83 ± 0.51	5.939	0.003*
Overall Test					
Between Groups (*F*,* P*) 112.18, 0.000*					
Time (*F*,* P*) 126.49, 0.000*					
Interaction (*F*,* P*) 36.186, 0.000*					

*p < 0.001

**Fig. 3:**
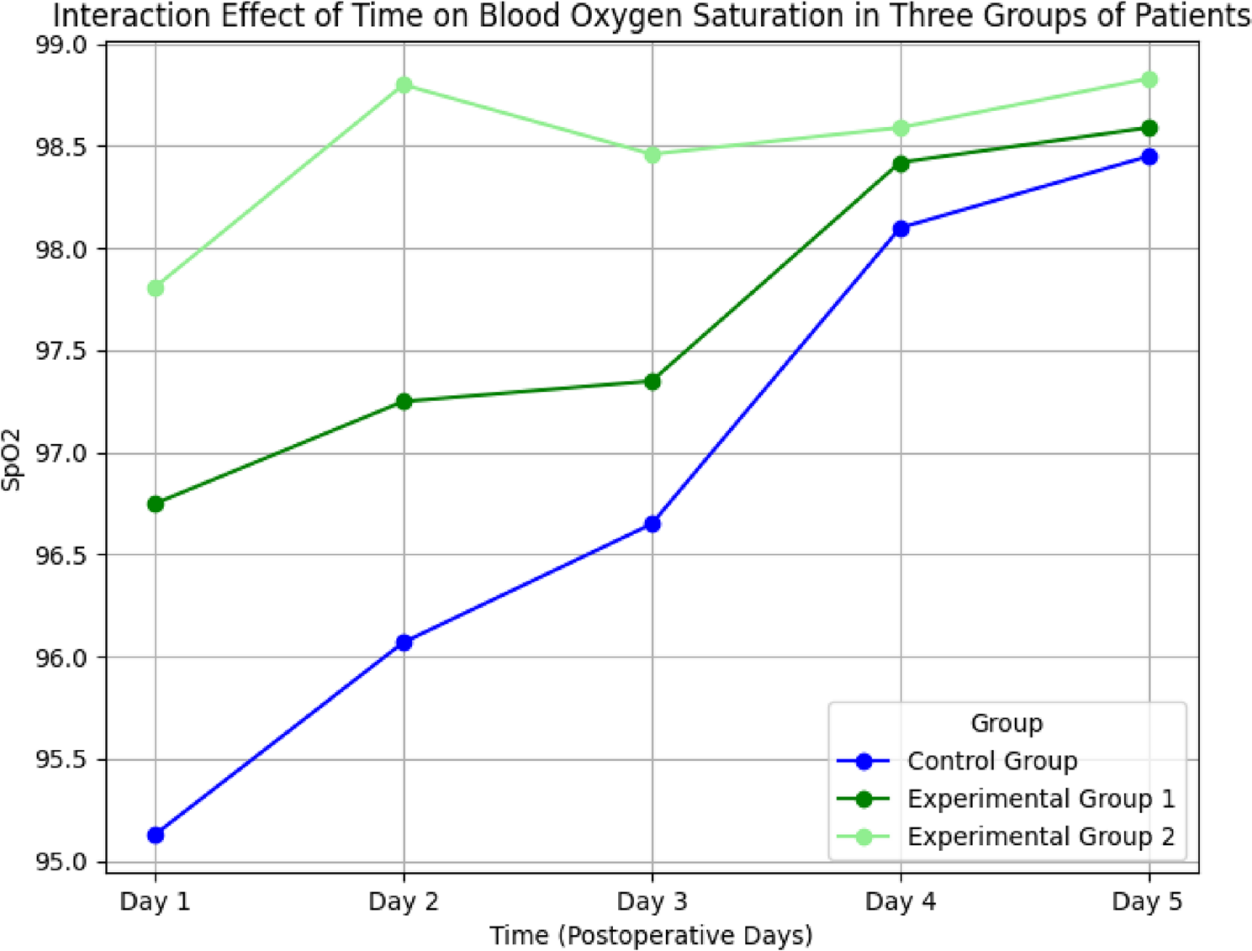
Three groups of patients' blood oxygen saturation changes over time interaction effect

The profile plot (Fig. 3) revealed that SpO₂ increased over time in all groups, with the highest levels observed in experimental group 2.

### Chest drainage tube retention and hospital stay

There were statistically significant differences among the three groups in both postoperative drainage tube retention time and length of hospital stay (*P* < 0.05) (Table 4).

**Table 4:** Comparison of postoperative chest drainage tube retention time, length of hospital stay, and satisfaction among the three groups (Mean ± SD)

Group	Sample Size (*n*)	Drainage Tube Retention Time (days)	Postoperative Hospitalization Duration (days)	Patient Satisfaction
Control Group	69	3.74 ± 2.83	6.42 ± 3.76	4.46 ± 0.50
Experimental Group 1	69	3.90 ± 3.25	6.39 ± 4.33	4.66 ± 0.48
Experimental Group 2	69	2.74 ± 1.11	4.99 ± 1.24	4.87 ± 0.34
*H*	-	10.638	7.868	26.121
*p*	-	0.005*	0.020*	0.000*

*Represents *p* < 0.05

Pairwise comparisons showed that experimental group 2 had significantly shorter retention time and hospital stay compared to both the control group and experimental group 1 (*P* < 0.05).

No significant differences were found between experimental group 1 and the control group (*p* > 0.05) (Table 5).

**Table 5:** Pairwise comparison of postoperative chest drainage tube retention time, length of hospital stay, and satisfaction among the three groups

Item	Group	Mean Difference	*p*
Chest Drainage Tube Retention Time (days)	Control Group vs. Experimental Group 1	−0.16	0.716
	Control Group vs. Experimental Group 2	1.00	0.023*
	Experimental Group 2 vs. Experimental Group 1	−1.16	0.009*
Length of Hospital Stay (days)	Control Group vs. Experimental Group 1	0.03	0.960
	Control Group vs. Experimental Group 2	1.43	0.014*
	Experimental Group 2 vs. Experimental Group 1	−1.41	0.016*
Satisfaction	Control Group vs. Experimental Group 1	−0.21	0.008*
	Control Group vs. Experimental Group 2	−0.41	0.000*
	Experimental Group 2 vs. Experimental Group 1	−0.21	0.007*

*p < 0.05

### Comparison of postoperative rehabilitation nursing satisfaction among the three groups

Significant differences in rehabilitation care satisfaction scores were observed among the three groups (*P* < 0.05) (Table 4).

Pairwise comparisons also indicated statistically significant differences between each group (*P* < 0.05) (Table 5).

## Discussion

### Position management combined with ACBT enhances sputum clearance and reduces pulmonary complications

Postoperative pulmonary complications (PPCs) are a major barrier to recovery after lung cancer surgery. Dai et al. [15] reported that pulmonary infections significantly increase postoperative mortality in lung cancer patients. Therefore, preventing or reducing PPCs is a critical goal in perioperative nursing. Effective clearance of airway secretions is essential to achieve this [16]. Respiratory training is a core component of postoperative pulmonary rehabilitation and plays a vital role in restoring lung function [17, 18]. ACBT, a structured breathing technique, has shown positive results in managing chronic respiratory diseases [19, 20]. However, it primarily targets airway clearance and may lack comprehensive physiological support. To address this, our study incorporated position management alongside ACBT. The results demonstrated that Group 2 (ACBT combined with position management) had a significantly lower incidence of PPCs compared to the control group (*P* < 0.05), aligning with findings by Lu et al. [21]. In their study, the application of ACBT reduced PPC incidence to 26%. In our trial, Group 2 had a PPC incidence of just 4.3%, compared with 13.0% in Group 1 (ACBT only) and 23.2% in the control group. This substantial difference highlights the added benefit of position management, which allowed patients to select body positions based on individual tolerance and comfort, thus enhancing secretion clearance.

For example, in our study:

- One patient with poor expectoration successfully cleared secretions using the prone position combined with ACBT.
- Two elderly patients unable to cough productively used a seated-forward position with ACBT, achieving effective sputum clearance.
- Several patients afraid to cough due to incisional pain used the lateral position (healthy side up, bed elevated at 60°) to facilitate expectoration.

These position-based adaptations not only improved sputum drainage but also reduced infection risk, consistent with Zhao et al. [22], who reported that position management decreases perioperative infection rates. Our results suggest that combining position management with ACBT has a synergistic effect, supporting secretion mobilization and pulmonary recovery more effectively than ACBT alone.

Furthermore, this combination was particularly effective in preventing atelectasis. Mechanistically, it promotes alveolar expansion, maintains chest wall mobility, strengthens respiratory muscles, and facilitates removal of intrapulmonary secretions. These factors collectively support lung re-expansion and restore function. Interestingly, our results contrast with those of Yang et al. [23], who reported no significant difference in PPCs between intervention and control groups using ACBT alone. The discrepancy may stem from the limited duration of their intervention (3 days), whereas our study applied the combined protocol for 5 days and customized it based on each patient’s condition. For instance, patients with weak cough strength benefited from prone positioning, which reduced dorsal lung compression, improved ventilation-perfusion balance, and enhanced airway drainage.

These findings underscore the clinical value of combining ACBT with individualized positioning strategies to reduce PPCs following thoracic surgery.

### Position management combined with ACBT improves oxygen saturation

Our study also found that the combination of ACBT and position management significantly improved SpO₂ levels postoperatively. This is consistent with findings by Zhang et al. [24], who noted that position changes can expand alveoli, improve lung ventilation, and reduce complications such as atelectasis and infection.

ACBT contributes by strengthening respiratory muscles and promoting effective expectoration, which facilitates optimal gas exchange. Position management complements this by enhancing baseline ventilation conditions. Together, they support maximal inspiratory and expiratory effort and help maintain efficient oxygenation.

Importantly, patients in Group 2 had consistently higher SpO₂ levels than those in Group 1, indicating that the combined intervention was more effective than ACBT alone. This suggests a synergistic relationship: position management optimizes lung mechanics, while ACBT engages patients in active participation, further improving ventilation dynamics and oxygen diffusion.

### Position management combined with ACBT reduces chest tube duration, hospital stay, and enhances rehabilitation satisfaction

Our findings indicate that the combination of position management and ACBT significantly shortens the duration of chest tube placement and hospital stay, while also improving patient satisfaction with rehabilitation care. These results suggest that the combined intervention not only accelerates postoperative recovery and reduces hospitalization costs, but also enhances patients’ perception of nursing quality. Both chest tube duration and hospital stay are Key indicators of recovery speed after thoracic surgery. The superior outcomes observed in Experimental Group 2 may be closely related to improvements in lung function and drainage efficiency achieved through the combined approach. Specifically, position management facilitates chest drainage by improving pulmonary ventilation and reducing atelectasis and fluid accumulation. Meanwhile, ACBT enhances negative intrathoracic pressure and supports mucus clearance through active patient participation. Together, these strategies restore alveolar tension and optimize the ventilation-perfusion ratio early in the recovery process. This synergistic effect leads to more efficient drainage of air and fluid from the chest cavity, thus reducing the time needed for chest tube placement. The resulting improvement in pulmonary function also contributes to earlier discharge readiness.

Early removal of the chest tube not only decreases the risk of postoperative infection and related complications but also encourages earlier ambulation and rehabilitation. In addition, ACBT requires active engagement from patients, which enhances their sense of control and adherence to rehabilitation. Reduced discomfort and stress from shorter chest tube duration and hospitalization improve the overall recovery experience and lead to higher patient satisfaction with nursing care.

### limitations

This study has several limitations that should be considered when interpreting the results. First, the study was conducted at a single tertiary center with a relatively small sample size (*n* = 69 per group), limiting statistical power and generalizability. Second, the intervention and follow-up were confined to the first five postoperative days, precluding assessment of long-term outcomes (e.g., late pulmonary complications, readmission, or functional recovery). Third, sputum volume, an objective indicator of airway clearance, was not quantitatively measured, which may weaken evidence of efficacy. Fourth, participants were not stratified by chronic obstructive pulmonary disease (COPD) or other respiratory comorbidities, which may influence response to respiratory training. Future research should employ multicenter designs with larger, stratified samples, incorporate objective measures (e.g., sputum volume, spirometry), quantitatively monitor adherence, and extend follow-up to evaluate sustained effects.

## Conclusions

In summary, this preliminary study suggests that position management combined with active cycle of breathing techniques (ACBT) may promote sputum clearance, potentially reduce postoperative pulmonary complications, shorten chest tube drainage duration and hospitalization, and improve oxygen saturation and patient satisfaction in lung cancer patients. While these findings indicate potential clinical value, the small sample size (*n* = 69) and single-center design limit their generalizability. Larger, multicenter trials are needed to validate these preliminary results and optimize intervention strategies. 

## Data Availability

The datasets generated and analyzed during the current study are available from the corresponding author upon reasonable request. As the original data will be used for subsequent research, they are not publicly available at this stage. Researchers requiring access to the original data for academic purposes may request it from the corresponding author after the article is accepted for publication. This approach ensures transparency and reproducibility while protecting the integrity of ongoing research.

## Abbreviations

ACBT: Active Cycle of Breathing Techniques
SpO₂: Peripheral Capillary Oxygen Saturation
NSCLC: Non-Small Cell Lung Cancer
PPCs: Postoperative pulmonary complications
